# Ambient Air Toxics and Asthma Prevalence among a Representative Sample of US Kindergarten-Age Children

**DOI:** 10.1371/journal.pone.0075176

**Published:** 2013-09-18

**Authors:** Alexis M. Stoner, Sarah E. Anderson, Timothy J. Buckley

**Affiliations:** 1 Division of Environmental Health Sciences, the Ohio State University College of Public Health, Columbus, Ohio, United States of America; 2 Division of Epidemiology, the Ohio State University College of Public Health, Columbus, Ohio, United States of America; UCL Institute of Child Health, University College London, United Kingdom

## Abstract

**Background:**

Criteria pollutants have been associated with exacerbation of children’s asthma, but the role of air toxics in relation to asthma is less clear. Our objective was to evaluate whether exposure to outdoor air toxics in early childhood increased asthma risk or severity.

**Methods:**

Air toxics exposure was estimated using the 2002 National Air toxics Assessment (NATA) and linked to longitudinal data (n=6950) from a representative sample of US children born in 2001 and followed through kindergarten-age in the Early Child Longitudinal Study - Birth Cohort (ECLS-B).

**Results:**

Overall, 17.7% of 5.5 year-olds had ever been told by a healthcare professional they had asthma, and 6.8% had been hospitalized or visited an emergency room for an asthma attack. Higher rates of asthma were observed among boys (20.1%), low-income (24.8%), and non-Hispanic black children (30.0%) (p≤0.05). Air toxics exposure was greater for minority race/ethnicity (p<0.0001), low income (p<0.0001), non-rural area (p<0.001). Across all analyses, greater air toxics exposure, as represented by total NATA respiratory hazard index, or when limited to respiratory hazard index from onroad mobile sources or diesel PM, was not associated with a greater prevalence of asthma or hospitalizations (p trend >0.05). In adjusted logistic regression models, children exposed to the highest respiratory hazard index were not more likely to have asthma compared to those exposed to the lowest respiratory hazard index of total, onroad sources, or diesel PM.

**Conclusions:**

Early childhood exposure to outdoor air toxics in a national sample has not previously been studied relative to children’s asthma. Within the constraints of the study, we found no evidence that early childhood exposure to outdoor air toxics increased risk for asthma. As has been previously reported, it is evident that there are environmental justice and disparity concerns for exposure to air toxics and asthma prevalence in US children.

## Introduction

About 7 million United States (U.S.) children have asthma and this represents a substantial public health burden. Air pollution has been demonstrated to exacerbate asthma but how much it does so and the strength of the evidence depends on the type of pollutant and extent of exposure [1,2]. Children are particularly susceptible to the harmful effects caused by air pollution because they spend more of their time outdoors (typically 50% more than adults), are more likely to participate in recreational activities that increase their ventilation and dose rates, and have a more a permeable respiratory epithelium [3,4,5].

Although criteria pollutants have been extensively studied and demonstrated to cause exacerbation of children’s asthma [5,6,7,8,9,10] the evidence with respect to air toxics is much less robust and clear. Air toxics (also known as hazardous air pollutants) are a class of 187 pollutants that are defined under Title 42 of the 1990 Clean Air Act Amendments that are distinct from the six criteria pollutants. Air toxics are known or suspected to cause cancer or other serious health effects, such as respiratory, reproductive, and birth defects. Example pollutants include benzene, perchloroethylene, dioxin, asbestos, toluene, and metals such as cadmium, mercury, chromium. They are emitted from a wide range of sources including mobile, industrial, area, and indoor. Air toxics are of public health concern because it has been estimated that fifty million people in the U.S. live in areas where concentrations are at levels that pose a health risk, however, very few studies have examined the relationship between that risk and actual disease including asthma [2].

Many air toxics have been linked to asthma, lung irritation, and other adverse respiratory effects but the evidence largely comes from animal or occupational studies at substantively higher concentrations than occurs from environmental exposures [11]. Environmental tobacco smoke (ETS) is independently associated with asthma prevalence among children [11,12,13], and of the 49 main components of ETS, twenty-nine are air toxics [12]. Additional evidence has been illustrated by many experimental and epidemiologic studies which have shown that exposure to polycyclic aromatic hydrocarbons in diesel exhaust contributes to allergic respiratory illness, and asthma exacerbation [14]. Evidence also indicates a respiratory irritant mechanism for many volatile organic compounds identified to be air toxics [14].

In 1996, the U.S. Environmental Protection Agency (EPA) conducted the first National Air Toxics Assessment (NATA). This assessment was designed to evaluate ambient (outdoor) air toxics in the U.S. in order to prioritize regulation and gain a better understanding of the public health risks [15]. NATA assessments provide model-based estimates of annual ambient concentrations of selected air toxics resulting from emissions by both stationary and mobile sources, along with estimated total cancer and non-cancer risks at the census tract resolution [16]. Annual estimates have been provided in three year intervals since 1996 to 2005. The next assessment will be for 2011 scheduled for release before the end of this year. Previous estimates from NATA suggest that air toxics exposures are widespread and in many areas and concentrations occur at levels that exceed benchmark levels for both non-cancer and cancer risk [17]. . Because air toxics are not routinely monitored, NATA modeled estimates provide one of the only ways to evaluate ambient exposures [18]. Studies suggest that while NATA modeled estimates have their limitations, they are a reasonable estimation for personal exposure to air toxics of ambient origin [19,20].

NATA was developed to assess air toxics ambient exposure and risk as a basis for public health protection. An important consideration is whether the estimated exposure and risk reflects actual disease risk. This has been explored for some outcomes including cancer [21], and autism spectrum disorders [18,22], however, to our knowledge, not children’s asthma. Accordingly, the goal of the current study is to investigate whether exposure to air toxics in early childhood is associated with prevalence of asthma at 5.5 years of age in a national sample of US children born in 2001. This is one of the most robust assessments examining the influence of outdoor air toxics on children’s asthma as previous studies have been limited in geographical scope and sample size [2,5,11].

## Methods

To explore possible associations between asthma prevalence among U.S. kindergarten-age children and exposure to air toxics, we conducted an analysis linking exposure estimates from NATA to ECLS-B children at the level of the zip code. We then determined if prevalence rates differed by air toxics risk quantified as a respiratory hazard index.

### Study Population

Prevalent asthma by zip code was derived from a longitudinal study conducted by the National Center for Education Statistics (NCES), to understand how factors in early childhood influence school readiness [23]. ECLS-B was funded and designed with input from multiple government agencies including the National Institute of Health, Office of Minority Health and Maternal and Child Health Bureau. ECLS-B employed a clustered list-frame design to sample births (n=14,000) from birth certificates maintained by the National Center for Health Statistics [24]. The study was designed to be representative of children born in the U.S. in 2001 to mothers 15 years and older. The sampling design has been previously described [24]. Enrollment and the first assessment occurred when children were approximately 9 months old (n=10,700) with follow-ups at 24 months (n=9850), 4.5 years (n=8750) and 5.5 years (Kindergarten-age, n=6950) [24]. The sample reduction at the 5.5 year wave was due in part to funding constraints that resulted in an 85% random selection. All assessments consisted of computer-assisted interviews with the child’s mother (usual) or father (rare) along with direct observation and measurement of children [25,26]. The NCES ethics review board approved the data collection, and parents provided written informed consent. The Ohio State University is licensed with the NCES for analysis of ECLS-B restricted-use data and we complied with NCES guidelines requiring that all sample sizes be reported with rounding to the nearest 50.

### Exposure to Air Toxics of Outdoor Origin

Children’s exposure to and respiratory risk from ambient air toxics was estimated from the 2002 U.S. EPA NATA assessment and linked to children’s residential zip code at the 24 month wave of ECLS-B. We used the child’s residential zip code at 24 months because it was within the time window of the 2002 NATA assessment. For the majority of children their residential zip code at 9 months and 24 months was the same. NATA provides estimates of ambient annual air toxics exposure for 180 pollutants at the census tract level. Because there is not a direct mapping of geographic areas defined by census tracks to postal zip codes, we used a commercially available database (Maponics^®^ 2006) to determine which census tracts (and the percentage of each) that were contained within a zip code [27]. To define each child’s ambient annual air toxics exposure we used a weighted average based on the percentage of the census tract area contained within the child’s zip code.

NATA is a model-based assessment of annual average concentrations and associated risk from ambient exposure to air toxics. Concentration estimates are developed based on the 2002 National Emission Inventory (NEI) coupled to meteorological data with an assumption of Gaussian air dispersion. Respiratory risk is a unitless value that is defined as a hazard quotient (HQ) and calculated as the ratio of the concentration estimate to the reference concentration (RfC) associated with respiratory risk. The RfC is defined as “an estimate (with uncertainty spanning perhaps an order of magnitude) of a continuous inhalation exposure of a chemical to the human population (including sensitive subpopulations), that is likely to be without risk of deleterious noncancer effects during a lifetime [17].” Accordingly, a HQ value >1.0 is indicative of a population at risk. RfC values for the 43 air toxics and associated with respiratory risk are provided in (Table 1). By convention, the unitless HQ values can be summed to give a hazard index (HI) that reflects the cumulative respiratory risk. In effect, the HI is the air toxic ambient concentration value weighted by toxicity and summed across all other air toxics known to pose a respiratory risk. In the context of this paper, the HI representing respiratory risk serves as the metric of air toxics exposure against which asthma prevalence and severity is compared. Because NATA is based on emission data, it is further possible to apportion the risk across five source categories including major stationary, area, on-road mobile, non-road mobile, and background. Detailed methods are described by the U.S. EPA [17].

**Table 1:** NATA 2002 Hazardous Air Pollutants Posing a Respiratory Risk and their Associated Reference Concentration.

**Chemical Name**	**RfC** ^ab^ **(µg/m** ^3^)	**Chemical Name**	**RfC** ^ab^ **(µg/m** ^3^)
Acetaldehyde	**9.0**	Hexamethylene-1,6-diisocyanate	**0.01**
Acrolein	**0.02**	Hydrochloric acid	**20.0**
Acrylic acid	**1.0**	Maleic anhydride	**0.7**
Acrylonitrile	**2.0**	Methyl bromide	**5.0**
Antimony Compounds	**0.2**	Methyl isocyanate	**1.0**
Beryllium Compounds	**0.02**	Methyl methacrylate	**700.0**
Bis(2-ethylhexyl) phthalate	**0.01**	Methylene diphenyl diisocyanate	**0.6**
Chlorine	**0.15**	Naphthalene	**3.0**
2-Chloroacetophenone	**0.03**	Nickel Compounds	**0.09**
Chloroprene	**2.0**	Nitrobenzene	**9.0**
Chromium Compounds	**0.1**	Phosgene	**0.3**
Cobalt Compounds	**0.02**	Phthalic anhydride	**20.0**
1,3-Dichloropropene	**20.0**	Propylene dichloride	**4.0**
Diesel Emissions	**5.0**	Propylene oxide	**30.0**
Diethanolamine	**3.0**	Styrene oxide	**6.0**
Epichlorohydrin	**1.0**	Titanium tetrachloride	**0.1**
1,2-Epoxybutane	**20.0**	Toluene	**5000.0**
Ethylene dibromide	**9.0**	2,4-Toluene diisocyanate	**0.07**
Ethylene glycol	**400.0**	Triethylamine	**7.0**
Formaldehyde	**9.8**	Vinyl acetate	**200.0**
Hexachlorocyclopentadiene	**0.2**		

^a^ RfC is derived from either IRIS (Integrated Risk Information system), CAL (California Environmental Protection Agency), or ATSDR (Agency for Toxic Substances and Disease Registry)

^b^ RfC is the reference level for which harmful health effects can occur as a results of inhalation exposure.

We considered the HI from three source categories. The first was the HI for all chemicals identified as posing a respiratory hazard from all sources, designated as HI_T_. The second HI similarly considered all of the Table 1 chemicals but was limited to only on-road mobile-sources (HI_MS_). The third HI was limited to ambient diesel particles from on-road mobile sources (HI_DPM_). We hypothesized that higher exposure to ambient air toxics during early childhood would be associated with increased asthma prevalence and severity at age 5.5 years. We looked separately at HI_MS_ and HI_DPM_ because previous studies have specifically implicated them in asthma exacerbation (reviewed in [14]).

### Asthma Prevalence and Severity

At each of the ECLS-B assessments (9 months, 24 months, 4.5 years, 5.5 years), a trained interviewer asked mothers, “Since your child turned (x) years of age, has a doctor, nurse, or other medical professional ever told you that your child has asthma?” We defined a child as having asthma if this question was answered affirmatively at any of the four assessments.

Asthma severity was also assessed. If the mother replied affirmatively that the child had asthma, she was asked, “Since your child turned (x) years of age how many times has a doctor, nurse, or other medical professional told you that your child had an asthma attack?” If the mother responded that the child had ≥ 1 asthma attack she was asked, “Since your child turned (x) years of age, has your child been taken to the emergency room (ER) or hospitalized for at least one night because of asthma?” We classified a child as having severe asthma if this final question was affirmatively answered at any of the four assessments.

### Covariates

Sociodemographic characteristics of each child were based on information collected during the 9 month (gender, race/ethnicity) and 24 month (income-to-poverty ratio, urbanicity, and maternal smoking status) assessments. Children that lived in an urban area or an urban cluster as defined by the 2000 U.S. census were classified as living in an urban dwelling [26]. Mothers were asked about their smoking practices and classified as: 1) nonsmoker; 2) smoker and smokes in the home; and 3) smoker but does not smoke in the home.

### Data Analysis

We restricted our analysis to children (n=6950) who were assessed at the kindergarten-age wave and whose residential zip code at 24 months could be linked to NATA estimates (<50 children lived in zip codes that could not be linked). Sampling weights that adjust for disproportionate sampling, non-response, and non-coverage were applied in our analyses and we used Jackknife replicate weights as implemented in the survey procedures in SAS 9.2 (SAS Institute, Inc, Cary, NC) to appropriately account for the clustered survey design in variance estimation and statistical testing [28].

We examined the distribution of each of the three NATA risk estimates (HI_T_, HI_MS,_ and HI_DPM_) among US kindergarten-age children, and constructed deciles based on the weighted data. Thus, the top decile indicates the 10% of children living in areas with the highest exposure to ambient air toxics as defined (i.e. HI_T_, HI_MS_, and HI_DPM_). We examined whether exposure to high HI_T_, HI_MS_, and HI_DPM_ were related to sociodemographic factors including gender, race/ethnicity, income to poverty ratio, urbanicity, and maternal smoking status. Statistical significance for these comparisons was based on an α level of 0.05 using design-adjusted likelihood ratio χ^2^ tests. Differences in asthma prevalence and hospitalization due to asthma were also examined in relation to these sociodemographic covariates.

We estimated the percentage of children within each decile of NATA HI_T_ who had been diagnosed with asthma by kindergarten-age and the percentage who had been hospitalized due to asthma. We used logistic regression to estimate the odds ratio for having been diagnosed with or hospitalized due to asthma across deciles of NATA HI_T_ where the lowest hazard index decile served as the reference. Odds ratios and 95% confidence intervals that are unadjusted as well as adjusted for child age, gender, race/ethnicity, income to poverty ratio, urbanicity, and maternal smoking status are presented. In these models, the decile of NATA HI_T_ was entered as a categorical variable. We also determined whether there was a linear trend between deciles of the three NATA risk estimates (HI_T_, HI_MS_, and HI_DPM_) and asthma by entering decile number (1-10) as a continuous variable. Statistical significance was based on an α level of 0.05 using design-adjusted likelihood ratio χ^2^ tests.

We conducted sensitivity analyses to determine whether our findings differed if we restricted the sample to children who had lived in the same zip code at both 9 months and 24 months (n=5400), if results differed if the sample was restricted to children who had lived at the same zip code across all four assessments (n=3400), as well as if the results differed when examining the outcome at 9 months, 24months, 4.5 years, or 5.5 years. We also examined whether the pattern of our findings differed by maternal smoking status.

## Results

Characteristics of the sample and population of U.S. children born in 2001 and assessed at age 5.5 years are presented in Table 2. When these children were 24 months old, fifteen percent lived in rural areas, and 19% lived with a mother who smoked. The proportion of boys and girls in the highest decile of NATA 2002 HI_T_, HI_MS,_ or HI_DPM_ did not differ (p > 0.05), but the risk was not evenly borne with respect to race/ethnicity, income to poverty ratio, or urbanicity (p<0.05). Children who were poorer or who were of minority race/ethnicity were more likely to be exposed to the highest decile across all three classes of respiratory hazard index. The highest respiratory hazard index was in urban areas. We also found that children whose mother’s smoked were less likely to be exposed to the highest decile of HI_T_, HI_MS,_ or HI_DPM_. This was primarily because within our study (and consistent with previous literature) the prevalence of smoking is highest in rural areas (data not shown) [29].

**Table 2:** Sociodemographic characteristics of children living in a zip code with the highest decile of NATA HI_T_, HI_MS_ and HI_DPM_

	**n^a^ (%**)	**High HI_T_^b^ %(95%CI**)	**P-Value^c^**	**High HI_MS_^b^ %(95%CI**)	**P-Value^c^**	**High HI_DPM_^b^ %(95%CI**)	**P-Value^c^**
**Total**	6950	10.0 (7.9, 12.1)		10.0 (7.6, 12.4)		10.0 (8.7, 11.4)	
**Gender**							
Female	3400 (48.8)	10.4 (7.8, 12.9)	0.53	10.8 (8.0, 13.5)	0.18	9.9 (8.3, 11.6)	0.87
Male	3500 (51.2)	9.7 (7.5, 11.9)		9.3 (6.7, 11.9)		10.1 (8.4, 11.8)	
**Racial-ethnic group**							
Hispanic	1250 (24.3)	20.9 (16.7, 25.2)	<0.0001	19.0 (15.1, 22.9)	<0.0001	20.1 (16.4, 23.9)	<0.0001
non-Hispanic black	1200 (15.4)	9.0 (4.4, 13.6)		12.2 (5.7, 18.7)		12.0 (9.4, 14.6)	
non-Hispanic white	2850 (53.7)	4.6 (3.2, 6.0)		4.8 (3.0, 6.6)		4.3 (3.1, 5.4)	
other, non-Hispanic	1600 (6.6)	16.4 (12.3, 20.4)		14.1 (10.7, 17.6)		15.2 (11.5, 19.0)	
**Income to Poverty Ratio**							
<1.0	1650 (23.2)	12.7 (8.9, 16.5)	0.005	13.7 (9.7, 17.8)	<0.0001	12.9 (10.2, 15.5)	<0.0001
>1.0 to 1.85	1550 (22.8)	11.0 (8.1, 13.8)		10.8 (8.1, 13.5)		12.3 (9.7, 14.8)	
>185% to 3.0	1950 (29.0)	8.8 (6.8, 10.8)		8.7 (6.1, 11.3)		8.4 (6.4, 10.4)	
> 3.0	1750 (25.0)	8.1 (5.6, 10.6)		7.4 (4.7, 10.0)		7.3 (5.5, 9.1)	
**Urbanicity**							
Urban	5800 (84.6)	11.8 (9.3, 14.2)	<0.0001	11.8 (9.0, 14.7)	NA	11.8 (10.3, 13.4)	<0.0001
Rural	1100 (15.4)	0.3 (0.0, 0.7)		0.0 (NA)		0.03 (0.0, 0.8)	
**Maternal Smoking at 24m**							
Smokes inside the house	600 (8.6)	2.3 (0.3, 4.4)	<0.0001	4.3 (1.7, 6.9)	0.002	4.1 (1.7, 6.6)	<0.0001
Smokes but not inside the house	700 (10.4)	6.2 (2.6, 9.8)		7.3 (3.2, 11.5)		3.8 (1.6, 6.0)	
Nonsmoker	5650 (80.9)	11.3 (9.1, 13.6)		11.0 (8.4, 13.5)		11.5 (9.9, 13.0)	

HI_T_ represents total respiratory hazard index, HI_MS_ represents respiratory hazard index from onroad mobile sources, and HI_DPM_ represents diesel PM. Information is missing for maternal smoking status for <10 children, diesel PM and onroad mobile sources for <50 children.

^a^ n are unweighted and are rounded to the nearest 50 to comply with restricted-use data requirements. Values may not total to 6950 due to rounding. Percentages and 95% CI are weighted.

^b^ High risk represents the highest decile of NATA total respiratory hazard index, diesel PM respiratory hazard index, and onroad mobile sources respiratory hazard index.

^c^ Rao-Scott likelihood ratio χ^2^ of difference between groups in high risk percentage.

At kindergarten-age, 17.7% of U.S. children born in 2001 had been reported to have asthma, and 6.8% of children had been taken to an ER or hospitalized at least once due to asthma (Table 3). Asthma prevalence and hospitalization differed by gender, race/ethnicity, income to poverty ratio, and maternal smoking (p<0.05). Boys, non-Hispanic blacks, poorer children, and children whose mothers smoked were more likely to have asthma, but asthma prevalence did not differ relative to urbanicity (Table 3).

**Table 3:** Percentage of children with asthma by sociodemographic characteristics.

	**Asthma % (CI**)**^a^**	**P value^b^**	**Asthma Hospitalization %(CI**)**^a^**	**P value^b^**
**Total**	17.7 (16.3, 19.1)		6.8 (6.0, 7.7)	
**Gender**				
Female	15.1 (13.4, 16.8)	<0.0001	5.4 (4.3, 6.5)	0.001
Male	20.1 (18.1, 22.1)		8.2 (6.9, 9.5)	
**Racial-ethnic group**				
Hispanic	17.2 (14.6, 19.8)	<0.0001	6.6 (4.2, 7.2)	<0.0001
non-Hispanic black	30.0 (26.5, 33.5)		14.3 (12.0, 16.6)	
non-Hispanic white	14.3 (12.5, 16.1)		5.3 (4.3, 6.4)	
other, non-Hispanic	18.4 (15.6, 21.1)		6.0 (4.0, 8.1)	
**Income to Poverty Ratio**				
<1.0	24.8 (21.9, 27.7)	<0.0001	10.2 (7.9, 12.5)	<0.0001
>1.0 to 1.85	18.1 (15.0, 21.2)		6.7 (5.3, 8.0)	
>185% to 3.0	16.4 (13.8, 19.1)		6.3 (4.9, 7.8)	
> 3.0	12.3 (10.2, 14.3)		4.5 (3.2, 5.8)	
**Urbanicity**				
Urban	17.6 (16.1, 19.1)	0.66	7.1 (6.2, 7.9)	0.34
Rural	18.3 (15.4, 21.3)		5.7 (3.3, 8.2)	
**Maternal Smoking at 24m**				
Smokes inside the house	22.5 (17.9, 27.1)	0.0003	10.2 (6.6, 13.7)	0.003
Smokes but not inside the house	23.5 (19.0, 28.1)		9.6 (6.5, 12.7)	
Nonsmoker	16.4 (15.0, 17.9)		6.1 (5.2, 7.0)	

^a^ Percentages and 95% CI are weighted.

^b^ Rao-Scott likelihood ratio χ^2^ of difference between groups in high risk percentage.

**Figure 1:**
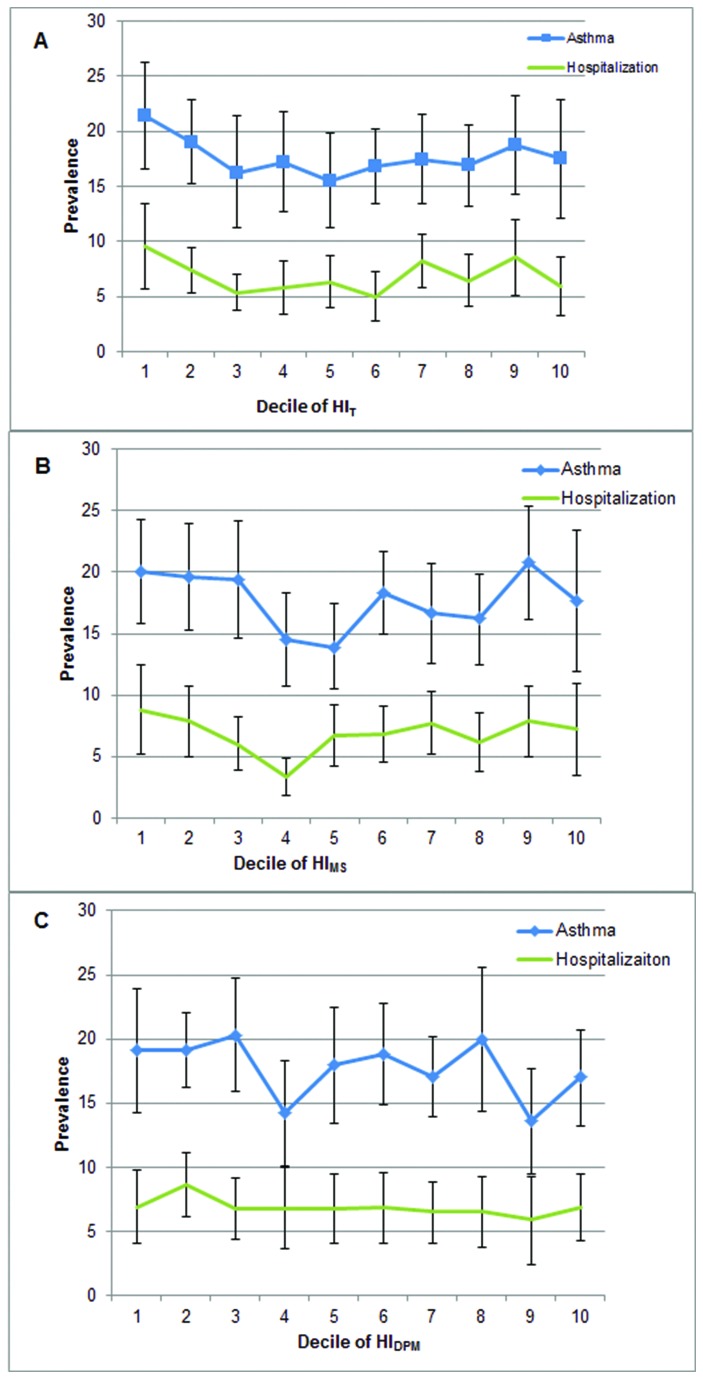
Prevalence and severity of asthma in relation to air toxics exposure. Prevalence of asthma and hospitalization due to asthma among U.S. kindergarten-age children across deciles of NATA respiratory hazard index (HI_T_) (panel A), onroad mobile sources (HI_MS_) (panel B), and diesel PM (HI_DPM_) (panel 3). Decile of air toxics exposure (1=low and 10=high) based on location of child’s residence at the 24 month assessment wave of ECLS-B. Prevalence estimates are weighted and represent the percentage of U.S. kindergarten-age children within each decile of air toxics exposure who had ever been diagnosed with asthma or hospitalized for asthma. Error bars are 95% confidence intervals.

Our hypotheses that higher exposure to ambient air toxics during early childhood would be associated with greater prevalence of children’s asthma or asthma hospitalization were not supported by the data. The prevalence of asthma or asthma hospitalization was not higher for children living in areas with greater HI_T_, HI_MS,_ or HI_DPM_. The prevalence (95% confidence interval) of asthma and hospitalization due to asthma for each decile of NATA respiratory hazard index is shown in the figure for each of the three respective risk estimates (Figure 1).

There was not a linear trend (p>0.05) across deciles of HI_T_ (Table 4), HI_MS_ (Table 5), HI_DPM_ (Table 6) and asthma prevalence or asthma hospitalization in unadjusted or adjusted logistic regression models. Compared to children living in a zip code with the lowest decile of HI_T_, children living with the highest HI_T_ were not significantly more likely to have been diagnosed with asthma [OR (95% CI): 0.68 (0.45-1.03)] or experience a hospitalization or ER visit due to asthma [OR (95% CI): 0.60 (0.36-0.99)] (Table 4). The odds of having asthma or being hospitalized due to asthma for children in the highest decile of HI_MS_ and HI_DPM_ were also not significantly different compared to children with the lowest decile of risk (Tables 5, 6). Our results did not differ substantively from those presented in tables 4-6 when we examined the association of HI exposure with asthma prevalence and hospitalization at each assessment wave separately. Findings were unchanged when restricted to children who had lived in the same zip code at 9 and 24 months, or who had lived in the same zip code from 9 months to kindergarten-age (not shown). Results did not differ relative to maternal smoking status (not shown).

**Table 4:** Asthma prevalence and severity among US kindergarten-age children in relation to their estimated HI_T_ exposure at 24 months.

		**Asthma**				**Hospitalization**		
**Decile**	**Respiratory HQ Range^c^**	**Prevalence %(CI**)**^e^**	**Unadjusted OR (95% CI**)**^d^**	**Adjusted^b^ OR (95% CI**)**^d^**		**Prevalence %(CI**)**^e^**	**Unadjusted OR (95% CI**)**^d^**	**Adjusted^b^ OR (95% CI**)**^d^**
1 (low)	0.04-<0.71	21.4 (16.6-26.2)	1.00 (referent)	1.00 (referent)		9.5 (5.7-13.4)	1.00 (referent)	1.00 (referent)
2	0.71 - <1.21	19.0 (15.2-22.8)	0.86 (0.59-1.27)	0.81 (0.57-1.15)		7.4 (5.3-9.4)	0.76 (0.44-1.30)	0.64 (0.39-1.03)
3	1.21- <1.95	16.2 (11.2-21.4)	0.72 (0.45-1.14)	0.70 (0.45-1.10)		5.3 (3.7-7.0)	0.54 (0.30-0.95)	0.46 (0.28-0.77)
4	1.95- <2.73	17.2 (12.7-21.8)	0.76 (0.50-1.17)	0.76 (0.51-1.13)		5.8 (3.4-8.2)	0.59 (0.30-1.16)	0.49 (0.27-0.91)
5	2.73- <3.56	15.5 (11.2-19.8)	0.67 (0.43-1.05)	0.65 (0.41-1.03)		6.3 (4.0-8.7)	0.64 (0.35-1.19)	0.50 (0.29-0.89)
6	3.56- <4.38	16.8 (13.4-20.2)	0.74 (0.51-1.08)	0.70 (0.48-1.02)		5.0 (2.8-7.3)	0.51 (0.26-0.97)	0.37 (0.21-0.65)
7	4.38- <5.18	17.4 (13.4-21.5)	0.78 (0.53-1.14)	0.68 (0.45-1.03)		8.2 (5.8-10.6)	0.85 (0.48-1.51)	0.60 (0.36-0.99)
8	5.18- <6.52	16.9 (13.2-20.6)	0.75 (0.51-1.10)	0.67 (0.45-1.00)		6.4 (4.1-8.8)	0.65 (0.36-1.19)	0.46 (0.27-0.81)
9	6.52- <8.94	18.7 (14.3-23.2)	0.85 (0.57-1.26)	0.75 (0.49-1.14)		8.6 (5.1-12.0)	0.89 (0.48-1.65)	0.63 (0.34-1.14)
10 (high)	8.94-26.29	17.5 (12.1-22.9)	0.78 (0.49-1.24)	0.71 (0.45-1.11)		5.9 (3.2-8.6)	0.60 (0.30-1.17)	0.47 (0.26-0.85)
P-Value (Test for Trend)^a^			0.50	0.34			0.66	0.21

**HI**_T_ represents total respiratory hazard index

^a^ P-value for test for trend: decile number modeled as a continuous variable in logistic regression (df = 1).

^b^ Adjusted for age, sex, race/ethnicity, income to poverty ratio, urbanicity, and maternal smoking status

^c^ NATA 2002 respiratory hazard quotient (HQ). Mean (95% CI) = 4.36 (4.10, 4.62)

^d^ Odds Ratio (95% confidence interval) from logistic regression model with decile modeled as a categorical variable

^e^ Percentages and 95% CI are weighted.

**Table 5:** Asthma prevalence and severity among US kindergarten-age children in relation to their estimated HI_MS_ exposure at 24 months.

		**Asthma**				**Hospitalization**		
**Decile**	**Onroad mobile range^c^**	**Prevalence %(CI**)**^e^**	**Unadjusted OR (95% CI**)**^d^**	**Adjusted^b^ OR (95% CI**)**^d^**		**Prevalence %(CI**)**^e^**	**Unadjusted OR (95% CI**)**^d^**	**Adjusted^b^ OR (95% CI**)**^d^**
1 (low)	0.00-<0.35	20.0 (15.8-24.3)	1.00 (referent)	1.00 (referent)		8.8 (5.2-12.5)	1.00 (referent)	1.00 (referent)
2	0.35- <0.71	19.6 (15.3-23.9)	0.97 (0.65-1.47)	0.94 (0.64-1.39)		7.9 (5.0-10.7)	0.89 (0.48-1.64)	0.82 (0.44-1.53)
3	0.71 - <1.19	19.4 (14.6-24.2)	0.96 (0.66-1.40)	1.00 (0.71-1.41)		6.0 (3.9-8.2)	0.67 (0.36-1.22)	0.64 (0.35-1.15)
4	1.19 - <1.69	14.5 (10.7-18.3)	0.68 (0.46-1.00)	0.67 (0.45-1.01)		3.4 (1.8-4.9)	0.36 (0.19-0.68)	0.32 (0.17-0.59)
5	1.69 - <2.30	13.9 (10.5-17.4)	0.65 (0.44-0.95)	0.70 (0.47-1.04)		6.7 (4.2-9.2)	0.75 (0.40-1.40)	0.70 (0.39-1.28)
6	2.30 - <2.95	18.3 (14.9-21.7)	0.89 (0.63-1.27)	0.90 (0.61-1.33)		6.8 (4.5-9.1)	0.76 (0.42-1.36)	0.63 (0.36-1.09)
7	2.95- <3.60	16.7 (12.6-20.7)	0.80 (0.54-1.18)	0.77 (0.50-1.18)		7.7 (5.2-10.3)	0.87 (0.48-1.57)	0.70 (0.39-1.25)
8	3.60- <4.32	16.2 (12.5-19.8)	0.77 (0.53-1.12)	0.74 (0.48-1.13)		6.2 (3.8-8.6)	0.68 (0.36-1.29)	0.54 (0.28-1.05)
9	4.32- <6.06	20.8 (16.1-25.4)	1.05 (0.73-1.52)	0.98 (0.67-1.42)		7.9 (5.0-10.7)	0.88 (0.49-1.60)	0.68 (0.37-1.26)
10 (high)	6.06-30.62	17.7 (11.9-21.4)	0.86 (0.54-1.37)	0.78 (0.47-1.30)		7.2 (3.5-10.9)	0.81 (0.39-1.68)	0,65 (0.31-1.38)
P-Value (Test for Trend)^a^			0.72	0.52			0.99	0.45

HI_MS_ represents respiratory hazard index from onroad mobile sources.

^a^ P-value for test for trend: decile number modeled as a continuous variable in logistic regression (df = 1).

^b^ Adjusted for age, sex, race/ethnicity, income to poverty ratio, urbanicity, and maternal smoking status

^c^ NATA 2002 onroad mobile sources respiratory hazard quotient (HQ). Mean (95% CI)=2.81 (2.62, 3.00).

^d^ Odds Ratio (95% confidence interval) from logistic regression model with decile modeled as a categorical variable

^e^ Percentages and 95% CI are weighted.

**Table 6:** Asthma prevalence and severity among US kindergarten-age children in relation to their estimated HI_DPM_ exposure at 24 months.

		**Asthma**				**Hospitalization**		
**Decile**	**Decile Range^c^**	**Prevalence %(CI**)**^e^**	**Unadjusted OR (95% CI**)**^d^**	**Adjusted^b^ OR (95% CI**)**^d^**		**Prevalence %(CI**)**^e^**	**Unadjusted OR (95% CI**)**^d^**	**Adjusted^b^ OR (95% CI**)**^d^**
1 (low)	0 -<0.035	19.1 (14.2-23.9)	1.00 (referent)	1.00 (referent)		6.9 (4.1-9.8)	1.00 (referent)	1.00 (referent)
2	0.035- <0.048	19.1 (16.2-22.0)	1.00 (0.68-1.47)	1.01 (0.68-1.49)		8.6 (6.1-11.1)	1.27 (0.73-2.19)	1.31 (0.76-2.24)
3	0.048- <0.063	20.3 (15.9-24.7)	1.08 (0.73-1.62)	1.10 (0.74-1.65)		6.8 (4.4-9.2)	0.98 (0.56-1.70)	0.95 (0.56-1.61)
4	0.063- <0.078	14.2 (10.0-18.3)	0.70 (0.44-1.12)	0.72 (0.46-1.12)		6.8 (3.6-10.1)	0.98 (0.47-2.04)	0.96 (0.48-1.92)
5	0.078-0.096	18.0 (13.4-22.5)	0.93 (0.61-1.42)	1.00 (0.66-1.52)		6.8 (4.1-9.5)	0.97 (0.52-1.84)	0.95 (0.53-1.71)
6	0.096- <0.114	18.8 (14.9-22.8)	0.99 (0.66-1.48)	0.97 (0.65-1.47)		6.9 (4.1-9.6)	0.99 (0.53-1.83)	0.85 (0.47-1.56)
7	0.114- <0.134	17.0 (13.9-20.2)	0.87 (0.60-1.27)	0.94 (0.64-1.38)		6.5 (4.1-8.8)	0.93 (0.51-1.71)	0.88 (0.48-1.61)
8	0.134- <0.166	20.0 (14.3-25.6)	1.06 (0.68-1.65)	1.05 (0.67-1.66)		6.5 (3.7-9.3)	0.93 (0.50-1.74)	0.79 (0.42-1.46)
9	0.166- <0.226	13.6 (9.5-17.7)	0.67 (0.42-1.07)	0.64 (0.40-1.04)		5.9 (2.4-9.3)	0.83 (0.38-1.84)	0.72 (0.33-1.59)
10 (high)	0.226-2.224	17.0 (13.2-20.7)	0.87 (0.58-1.30)	0.81 (0.54-1.23)		6.9 (4.3-9.5)	0.99 (0.54-1.82)	0.87 (0.49-1.54)
P-Value(Test for Trend)^a^			0.25	0.21			0.40	0.12

HI_DPM_ represents respiratory hazard index from diesel PM.

^a^ P-value for test for trend: decile number modeled as a continuous variable in logistic regression (df = 1).

^b^ Adjusted for age, sex, race/ethnicity, income to poverty ratio, urbanicity, and maternal smoking status

^c^ NATA 2002 Diesel PM. Mean (95% CI) = 0.118 (0.111, 0.124).

^d^ Odds Ratio (95% confidence interval) from logistic regression model with decile modeled as a categorical variable

^e^ Percentages and 95% CI are weighted

## Discussion

Relying on a nationally representative sample of US children born in 2001 and assessed from infancy through 5.5 years of age, we found no evidence that early childhood exposure to ambient air toxics as estimated by NATA was associated with increased risk for asthma or increased severity of asthma. To our knowledge, this is the largest and first study to use NATA to investigate the role of air toxics in pediatric asthma prevalence. Our observed results do not support the suggested link of ambient air toxics exposure and asthma prevalence in young children. The current study is unique in size (n=6950), national scale, population-based representative sampling, and consideration of multi-pollutant exposure/risk.

Based on our hypotheses, we expected to find higher prevalence of asthma and asthma hospitalization for children who lived in areas with high levels of ambient air toxics. Our results did not support this hypothesized association and in fact were suggestive of a negative or protective association. In these data, we observed the highest prevalence of asthma and asthma hospitalization for children exposed to the lowest respiratory HI. We are unaware of a biological basis to explain this observation. It is possible that families with children at risk for asthma move to locations where the air quality is better. It is also possible that bias or residual confounding are responsible for the observed results.

Previous studies have shown a strong positive association between prevalent asthma and the criteria pollutants [5,6,7,9,10]. While evidence of a correlation has been suggested, the role of ambient air toxics in asthma prevalence has not been as well studied and is less clear [11,14]. There is also limited suggestive evidence of asthma exacerbation as a result of air toxics exposure [11,14,30,31,32]. Both epidemiological and experimental studies have implicated exposure to polycyclic aromatic hydrocarbons (PAH), volatile organic compounds, ETS, and occupational and industrial exposures with increased risk for asthma. Chronic respiratory symptoms were positively associated with VOC exposure including emissions from chemical manufacturing plants among children [32]. Among asthmatic children, increased exposure to PAH was associated with an increased odds of wheeze [30]. Also, children who were exposed to higher prenatal PAH concentrations or postnatal ETS concentrations were more likely to develop asthma and other respiratory illness by 24months of age [31].

Childhood asthma prevalence is increasing in the United States. In 2005, 8.6% of children ages 0-4 and 13.7% of children ages 5-14 had been diagnosed with asthma [33]. We observed a higher prevalence of asthma (17.7%) among U.S. kindergarten-age children. There may be a number of reasons for this. We chose to classify a child as having been diagnosed with asthma if their parent reported at 9 months, 24 months, 4.5 years or 5.5 years that a doctor or other medical professional had told them their child had asthma. Asthma diagnosis is often difficult before the age of 5 years and for many children symptoms decrease with increasing age [34]. Children often cough or wheeze when they present with colds or viral infections and because there is no diagnostic test for asthma in young children, this wheezing can be mistaken for asthma [34]. Recent trends have shown a disproportionate increase in asthma prevalence in children less than five years of age [35]. Because children in ECLS-B were studied from infancy through kindergarten-age, it is probable that there may be an over diagnosis of asthma due to lack of consistent and transparent symptoms in early childhood along with an increase in diagnostic awareness among physicians and parents [36].

As has been observed by others [37], we observed that children of color, low income, and living in urban environments bore a disproportionate burden of air toxics exposure and risk substantiating an air toxics environmental justice concern that has been previously shown for cancer [38,39], child development [40], and asthma [41]. Also, as expected and previously reported, we observed substantially higher asthma prevalence rates among minority (especially non-Hispanic black) children, those from low income homes, and those who have a mother who smokes [33]. Minority populations of lower socio-economic status tend to live in areas where more physical, environmental, and economic stresses occur, e.g. more crowded homes, poor quality housing, and greater exposure to environmental toxics [37]. Therefore, higher air pollution concentrations and resulting adverse health effects in people of all ages are commonly disproportionately found in minorities and people with lower income to poverty ratios [42,43]. Our observation of lower smoking prevalence rates among urban households supports previous reports. National survey data indicates 27.8 percent of rural adults are smokers compared to 22.7 of urban residents [29]. Additionally, rural young adults are 27 percent more likely to smoke compared to those living in an urban residential area [29].

Reliance on NATA for estimating exposure has both significant strengths and limitations. NATA has been extensively peer-reviewed and has been the basis of studies that support its validation against measured values [16,19,20,38]. NATA provides extensive geographic coverage that would not be feasible if direct measurement was used. Furthermore, NATA is advantageous in providing a means to consider multiple air toxics (cumulative exposure and risk) over a relatively long period of time (i.e. annual average) as an appropriate “chronic” exposure metric for a disease outcome such as asthma. Also, NATA risk can be parsed by various source categories having direct relevance for informing mitigation. These same features of NATA that convey advantage, also present limitations. The modeling system used by the EPA known as the Assessment System for Population Exposure Nationwide, has been found to underestimate measurements [17,18]. NATA is also heavily weighted based on the National Emissions Inventory (NEI). Not all sources of pollutants report to the NEI and reporting is based on self-reports that may be unreliable [17,18]. When data are missing due to under reporting or lack of information such as stack height or facility location, default assumptions are made [17,18]. These limitations can contribute to exposure misclassification.

Because the models and inputs that underlie the NATA estimates have evolved under rigorous scientific scrutiny, and measurement validation studies have shown strong agreement [19,20], these limitations are somewhat known and quantified. However, NATA only considers air toxics from outdoor sources, and thus our study only assessed this aspect of children’s exposure to air pollution. We had limited information about children’s exposure to secondhand smoke, which is a critical determinant of indoor air toxics concentrations. We observed that prevalence and severity of children’s asthma was higher when mothers reported being current smokers. However, in comparison to the criteria pollutants that have been linked to asthma prevalence, air toxics occur in much smaller concentrations in the atmosphere and higher concentrations indoors [2]. Studies have shown that people are most commonly exposed to air toxics during their indoor activities and the indoor environment is of greater significance than the outdoor in relation to air toxics exposure [2,19,42,44]. While indoor air concentrations can often be highly variable and difficult to monitor over long periods and large scale, there is no doubt that it is an important component to examine when studying the health effects of air toxics and one that our current study does not address.

An additional limitation to these analyses lies within the fact that while this study represents a longer exposure assessment time period than most studies, it does not include prenatal exposure. While less is known about prenatal air toxics exposure, prenatal exposure to criteria pollutants can adversely affect lung growth and development in early childhood, especially among children with asthma and therefore should be considered in future analyses.

## Conclusions

This research demonstrates the utility of NATA to examine air toxics ambient exposure in a nationally representative sample. It is evident from previous research that criteria pollutants play an important role in the severity and exacerbation of asthma symptoms. Within the constraints of the current study, we saw no influence of ambient air toxics exposure on the prevalence of asthma in young children.

A clear and compelling environmental justice concern has been identified with children of color and who are socioeconomically disadvantaged bearing a disproportionate burden of respiratory air toxics risk. Even in the absence of evidence of an association between risk and prevalent disease, identification of this environmental injustice warrants attention.
